# Impact of High-Intensity-NIV on the heart in stable COPD: a randomised cross-over pilot study

**DOI:** 10.1186/s12931-017-0542-9

**Published:** 2017-05-02

**Authors:** Marieke Leontine Duiverman, Petra Maagh, Friederike Sophie Magnet, Claudia Schmoor, Maria Paola Arellano-Maric, Axel Meissner, Jan Hendrik Storre, Peter Jan Wijkstra, Wolfram Windisch, Jens Callegari

**Affiliations:** 1 Department of Pulmonary Diseases, University of Groningen, University Medical Center Groningen, Groningen, The Netherlands; 2 Groningen Research Institute of Asthma and COPD (GRIAC), University of Groningen, Groningen, The Netherlands; 3 Cologne Merheim Hospital, Department of Pneumology, Kliniken der Stadt Köln gGmbH Witten/Herdecke University, Faculty of Health/School of Medicine, Köln, Germany; 4 Department of Cardiology, Cologne Merheim Hospital, Kliniken der Stadt Köln gGmbH Witten/Herdecke University, Faculty of Health/School of Medicine, Köln, Germany; 5 Clinical Trials Unit, Faculty of Medicine and Medical Center - University of Freiburg, Freiburg, Germany; 6 Department of Pulmonary Diseases, Pontificia Universidad Católica de Chile, Santiago, Chile; 7 Asklepios Fachkliniken Munich-Gauting, Gauting, Germany; 8 Department of Pneumology, University Medical Hospital, Freiburg, Germany

**Keywords:** Chronic obstructive pulmonary disease, Non-invasive ventilation, High-intensity non-invasive ventilation, Cardiac output, Echocardiography, Gas exchange, Lung Function, Health-related Quality of Life, Respiratory muscle activity

## Abstract

**Background:**

Although high-intensity non-invasive ventilation has been shown to improve outcomes in stable COPD, it may adversely affect cardiac performance. Therefore, the aims of the present pilot study were to compare cardiac and pulmonary effects of 6 weeks of low-intensity non-invasive ventilation and 6 weeks of high-intensity non-invasive ventilation in stable COPD patients.

**Methods:**

In a randomised crossover pilot feasibility study, the change in cardiac output after 6 weeks of each NIV mode compared to baseline was assessed with echocardiography in 14 severe stable COPD patients. Furthermore, CO during NIV, gas exchange, lung function, and health-related quality of life were investigated.

**Results:**

Three patients dropped out: two deteriorated on low-intensity non-invasive ventilation, and one presented with decompensated heart failure while on high-intensity non-invasive ventilation. Eleven patients were included in the analysis. In general, cardiac output and NTproBNP did not change, although individual effects were noticed, depending on the pressures applied and/or the co-existence of heart failure. High-intensity non-invasive ventilation tended to be more effective in improving gas exchange, but both modes improved lung function and the health-related quality of life.

**Conclusions:**

Long-term non-invasive ventilation with adequate pressure to improve gas exchange and health-related quality of life did not have an overall adverse effect on cardiac performance. Nevertheless, in patients with pre-existing heart failure, the application of very high inspiratory pressures might reduce cardiac output.

**Trial registration:**

The trial was registered in the Deutsches Register Klinischer Studien (DRKS-ID: DRKS00007977).

**Electronic supplementary material:**

The online version of this article (doi:10.1186/s12931-017-0542-9) contains supplementary material, which is available to authorized users.

## Background

Chronic obstructive pulmonary disease (COPD) is a condition with high mortality and morbidity rates worldwide [1, 2]. Patients with end-stage COPD frequently develop chronic hypercapnic respiratory failure, at which point treatment options become limited.

The use of long-term nocturnal non-invasive ventilation (NIV) in COPD patients with chronic hypercapnic respiratory failure remains controversial [3–7]. However, since the application of high-intensity-NIV (HI-NIV) with higher inspiratory positive airway pressures (IPAP) and higher back-up respiratory rates (BURR), substantial benefits of chronic NIV have been reported [8–10].

Nevertheless, mechanical ventilation can affect cardiac output (CO), especially if it is applied with higher inspiratory and/or expiratory positive airway pressures (EPAP) [11–14]. A recent physiological study showed that HI-NIV in stable COPD acutely reduces CO [11]. However, positive effects of HI-NIV [11] compared to a setting with a lower IPAP and low BURR (low-intensity NIV (LI-NIV)), such as reduction in work of breathing and improvement in gas exchange, might turn the effects on the heart beneficial [14].

Long-term effects of NIV on the heart in COPD patients have so far only been sparsely investigated. Although long-term NIV was shown to increase heart rate variability [15, 16], and tended to decrease N-terminal brain natriuretic peptide (NTproBNP) levels [17], no studies have investigated long-term effects of chronic HI-NIV on cardiac performance in COPD patients with chronic hypercapnic respiratory failure and compared these effects according to the applied ventilatory mode. Therefore, the present pilot study aims to compare the effects of 6-week LI-NIV versus 6-week HI-NIV on cardiac and pulmonary function in COPD patients with chronic hypercapnic respiratory failure. To investigate cardiac performance we made use of echocardiography and analysed the feasibility of this technique to measure cardiac output in patients with severe COPD.

## Methods

### Patients

COPD patients with Global initiative of Obstructive Lung Diseases stage III or IV [1], and with an indication for chronic NIV were included. According to the German guidelines, COPD patients with symptoms of chronic respiratory failure are indicated for chronic NIV once they have: a) ≥ 2 severe acute exacerbations with acute hypercapnic respiratory failure (pH < 7.35); b) and/or a daytime partial arterial carbon dioxide (PaCO_2_) level ≥ 6.7 kilopascal (kPa); c) and/or a nocturnal PaCO_2_ ≥ 7.3 kPa or a rise in transcutaneous carbon dioxide pressure (P_t_CO_2_) during the night ≥1.3 kPa (doi:10.1055/s-0029-1243978). Patients had to be in stable condition (for at least 4 weeks without exacerbation, pH >7.35). Patients with neuromuscular and restrictive thoracic diseases or obstructive sleep apnoea were excluded.

The study protocol was approved by the Medical Ethics Committee of the University Witten Herdecke. Written informed consent was obtained from all subjects. The trial was registered in the Deutsches Register Klinischer Studien (DRKS-ID: DRKS00007977).

### Study design

The study was designed as a pilot feasibility study with a randomised, open-label, two-treatment, two-period crossover design. Patients were randomized to receive either the HI-NIV/LI-NIV or the LI-NIV/HI-NIV sequence. After baseline measurements were done, NIV was titrated in-hospital. Thereafter, patients were discharged, re-admitted after 6 weeks of home NIV to perform outcome measurements, and switched to the complementary mode of NIV. After another 6 weeks of home NIV, patients were re-admitted for final measurements. The study was a single-centre study at the Department of Pneumology, Merheim Hospital Cologne, Germany.

The primary outcome was the percentage change in CO during spontaneous breathing after 6 weeks of NIV compared to baseline. We investigated whether it was feasible to assess CO both during spontaneous breathing and NIV by echocardiography in our patients with severe COPD.

### Measurements

#### Cardiac performance

A complete standard M-mode and two-dimensional echocardiography was performed by one operator using a Philips iE33 ultrasound system with a S5-1 sector array probe (Philips GmBH, Hamburg, Germany). We assessed cardiac function by using transthoracic echocardiography because quantification of cardiac function is the cornerstone of cardiac imaging, with echocardiography being the most commonly used non-invasive modality because of its unique ability to provide real-time images of the beating heart, combined with its availability and portability.

Measurements were carried out on three representative beats and results were averaged. Heart rate (HR) was recorded. The following parameters were evaluated: (http://asecho.org/guidelines/guidelines-standards/) [18].

Left heart systolic function was assessed by Simpson’s rule: Left ventricle (LV) ejection fraction (LVEF) = (end-diastolic volume - end-systolic volume)/end-systolic volume. For LV CO, the left ventricular outflow tract (LVOT) diameter was measured at the level of the aortic annulus from the parasternal long-axis view in midsystole [19]. After that, the LVOT area was calculated as ∏ (LVOT diameter/2)^2^. LVOT Doppler pulsed-wave measurements were obtained over 3 consecutive cardiac cycles. The pulsed sample volume was placed in the LVOT in the anteriorly angulated 4-chamber apical view just proximal to the aortic valve to record the maximum flow signal. Proper location in the LVOT was confirmed by the aortic valve closure signal. After these measurements the diameter of the aortic annulushis was then multiplied by the velocity time integral of the trace of the Doppler flow profile across the aortic valve to determine the flow volume per beat (stroke volume, SV). The result was then multiplied by the HR to obtain CO.

For the right heart size and systolic function we measured right ventricle (RV) size, RV subcostal wall thickness, systolic pulmonary artery pressure (sPAP) with an estimate of right atrial pressure on the basis of inferior vena cava size and collapse, tricuspid annular plane systolic excursion (TAPSE), and fractional area change (FAC) and S´ (the pulsed Doppler peak velocity at the tricuspid annulus).

These parameters were assessed at each visit during (i) spontaneous breathing, and (ii) after 10 min acclimatization time, whilst undergoing NIV. Furthermore, 24-h ECG and blood pressure measurements were performed to detect rhythm disturbances and monitor blood pressure respectively, whilst on NIV. NTproBNP was assessed by routine laboratory methods.

Echocardiographic studies were performed in the standard left decubitus position by a single cardiologist, blinded to clinical and laboratory information. After optimizing image quality, stroke volume was measured from the anteriorly angled apical four-chamber view or three chamber view using pulsed wave Doppler with the interrogation beam directed across the LVOT. For the LVOT diameter, the zoom was used in the parasternal long axis view in systole to minimize the errors in the measurement. It is known that the LVOTdiameter is normally related to body surface area and should be around 20 mm for an average size person.

#### Pulmonary outcomes

The effectiveness of NIV was assessed by arterial (ABL 800 flex®, Firma Radiometer, Denmark) and transcutaneous blood gases (SenTec Digital Monitoring System®; SenTec AG; MPB-Software®: V05.03.02, SMB-Software®: V07.03.1; SenTec AG; Therwil, Switzerland) [20, 21]. Furthermore, lung function [22–24] and the 6-min walking test was performed [25] Surface electromyography (EMG; Dipha®-16 (DEMCON - Macawi Respiratory Systems BV, Enschede, the Netherlands)). was used to assess respiratory neural drive, as a surrogate parameter of work of breathing [26, 27]. HRQoL was measured with the Severe Respiratory Insufficiency Questionnaire (SRI) [28] and the COPD assessment test (CAT) [29]. Further details are displayed in Additional file 1. Compliance with NIV was assessed by the ventilator counter reading.

### Ventilatory modes

HI-NIV was titrated to establish normocapnia, or the lowest arterial carbon dioxide (PaCO_2_) value possible, according to the patient’s tolerance [30, 31]. For HI-NIV, an assist control mode with a BURR just above the spontaneous breathing frequency during sleep was used. IPAP was titrated at the highest tolerable level with an optimal reduction in PaCO_2_, EPAP was set at 4–6 cm H_2_O. For LI-NIV, pressure support ventilation with an IPAP ≤ 14 cm H_2_O and BURR of ≤ 12 breaths/min was used. During LI-NIV titration, an IPAP of up to 18 cm H_2_O and a BURR of up to 14 breaths/min were allowed for patient tolerance. The IPAP difference between the LI-NIV and HI-NIV setting had to be at least 4 cm H_2_O. Nasal and oronasal masks were used and not changed during the study. For further details, see Additional file 1.

### Statistical analysis

#### Sample size

At least 12 patients were needed in order to detect a clinically relevant change of at least 10% is present (with a standard deviation of 10%) in CO between LI-NIV and HI-NIV compared to baseline, at a two-sided level alpha of 0.05 with a power of 88% [11]. For an expected maximal drop-out rate of 20%, 14 patients needed to be included.

#### Statistical analysis

Comparison of LI-NIV and HI-NIV in the crossover setting was performed in the full analysis set, which included all randomised patients who received both treatments. Outcome measurements were tested for normality using visual inspection. An analysis of variance model was used with “treatment”, “period” and “randomised sequence” defined as fixed effects, and “patient within sequence” defined as a random effect. The treatment effect was estimated with a 95% CI and tested with a two-sided level of 0.05. In addition, tests for periodic and carryover (treatment-period interactions) effects were performed. Treatment result and baseline measurement were compared by calculating the mean difference with 95% CI and performing a paired *t*-test separately for LI-NIV and HI-NIV for all patients who received the respective treatment.

## Results

### Patient characteristics

Nine patients had arterial hypertension, three a reduced LVEF (50%, 20% and 25%), one reported previous ischemic heart disease with a percutaneous coronary intervention 2 years earlier, one had atrial fibrillation, two peripheral vascular disease, two hypercholesterolemia and one type 2 diabetes mellitus. All patients used long-acting beta-agonists, ten patients long-acting anticholinergics, seven inhaled corticosteroids, one roflumilast and one theophylline. None of the patients used oral corticosteroids. No medication changes were made during the follow-up (Table 1).

**Table 1:** Baseline characteristics of all patients

ID	Age yrs	Sex	BMI kg/m^2^	PaCO_2_ day, kPa	PaO_2_ day, kPa	HCO_3_, day, mmol/L	PaCO_2_ night, kPa	Δ PtCO_2_ night, kPa	LTOT l/min	FEV_1_ %pred	FEV_1_/FVC, %
1	57	M	34.7	5.8	8.8	27.0	7.6	2.5	-	23	34
2	74	F	28.0	6.3	9.3	28.6	7.0	2.8	2.0	39	52
3	53	M	21.4	6.1	7.7	28.3	6.5	1.3	-	28	38
4	70	F	27.2	6.4	8.4	27.2	6.8	5.3	4.0	35	68
5	67	M	35.9	7.1	9.3	36.0	7.3	2.2	-	50	66
6	82	M	21.5	8.6	5.9	31.9	10.6	0.8	2.0	30	63
*7*	*78*	*F*	*31.6*	*7.6*	*7.8*	*30.6*	9.6	1.5	*2.5*	*29*	*51*
8	74	F	22.5	7.8	10.9	30.6	8.0	2.5	3.0	36	61
9	68	M	19.4	7.3	8.8	28.6	6.9	2.7	4.0	36	40
10	63	F	22.8	7.4	8.6	29.3	8.2	1.3	3.0	18	39
11	71	F	25.1	5.9	9.6	27.0	7.4	2.6	1.0	35	66
*12*	*63*	*F*	*27.8*	*8.1*	*8.7*	*28.4*	8.8	4.6	*3.0*	*37*	*61*
*13*	*60*	*F*	*18.3*	*9.4*	*7.1*	*38.5*	8.8	1.9	*3.0*	*11*	*38*
14	77	F	18.7	6.4	10.2	28.0	7.5	1.0	2.0	41	49
Mean	68.7		25.2	6.8	8.9	29.3	7.0	2.3		34	53
SD	8.5		5.8	0.9	1.3	2.7	2.5	1.2		9	13

ID 7, 12 and 13 (italic) were excluded from analysis as they dropped out of the study. The mean and standard deviation (SD) of the 11 patients included in the full data set analysis are shown

*BMI* body mass index, *PaO*_*2*_ partial arterial oxygen pressure in kilopascal (kPa), *PaCO*_*2*_ partial arterial carbon dioxide pressure in kPa, *HCO*_*3*_^*−*^ bicarbonate, *Δ PtCO*_*2*_ increase in transcutaneous carbon dioxide pressure measured continuously at the ear lobe during the night; all blood gasses measured without NIV and with the regular amount of oxygen prescribed

*FEV*_*1*_ forced expiratory volume in 1 s, *FVC* forced vital capacity

### Ventilatory settings

Ventilatory settings and compliance rates of the study completers are shown in Table 2.

**Table 2:** Ventilatory settings, days needed to initiate NIV, and compliance rates of the study completers

Ventilatory setting	LI-NIV	HI-NIV
IPAP, cm H_2_O, mean ± SD	15.5 ± 1.1	23.6 ± 3.1
EPAP, cm H_2_O, mean ± SD	5.2 ± 0.6	5.4 ± 0.9
BURR, breaths/min, mean ± SD	11.6 ± 1.5	15.4 ± 0.8
Supplemental oxygen, L/min, mean ± SD	1.6 ± 1.3	1.4 ± 1.2
Days needed to initiate NIV, median (range)	2.7 (2–5)	4.9 (2–10)
Number of hours used/day, median (range)	4.2 (0.04–7.5)	4.6 (0.11 to 9.2)
Percentage of days that NIV was used, % (range)	63 (2–100)	79 (2–100)
Mask, nasal, n (%))/oronasal, n (%))	3 (27)/8(73)	
Exhalation, vented mask (n (%))/active valve (n (%))	9 (82)/2 (18)	
Humidification, n (%)	10 (90)	

*IPAP* inspiratory positive airway pressure, *EPAP* expiratory positive airway pressure, *BURR* backup respiratory rate set on the ventilator, *Supplemental oxygen* connected to the ventilator, *LI-NIV* low-intensity NV, *HI-NIV* high-intensity NIV

Patient ID 12 and 13 only completed the HI-NIV period and were ventilated with higher IPAP (IPAP 31 and 28 cm H_2_O), but similar BURR

### Drop-outs

**Fig. 1:**
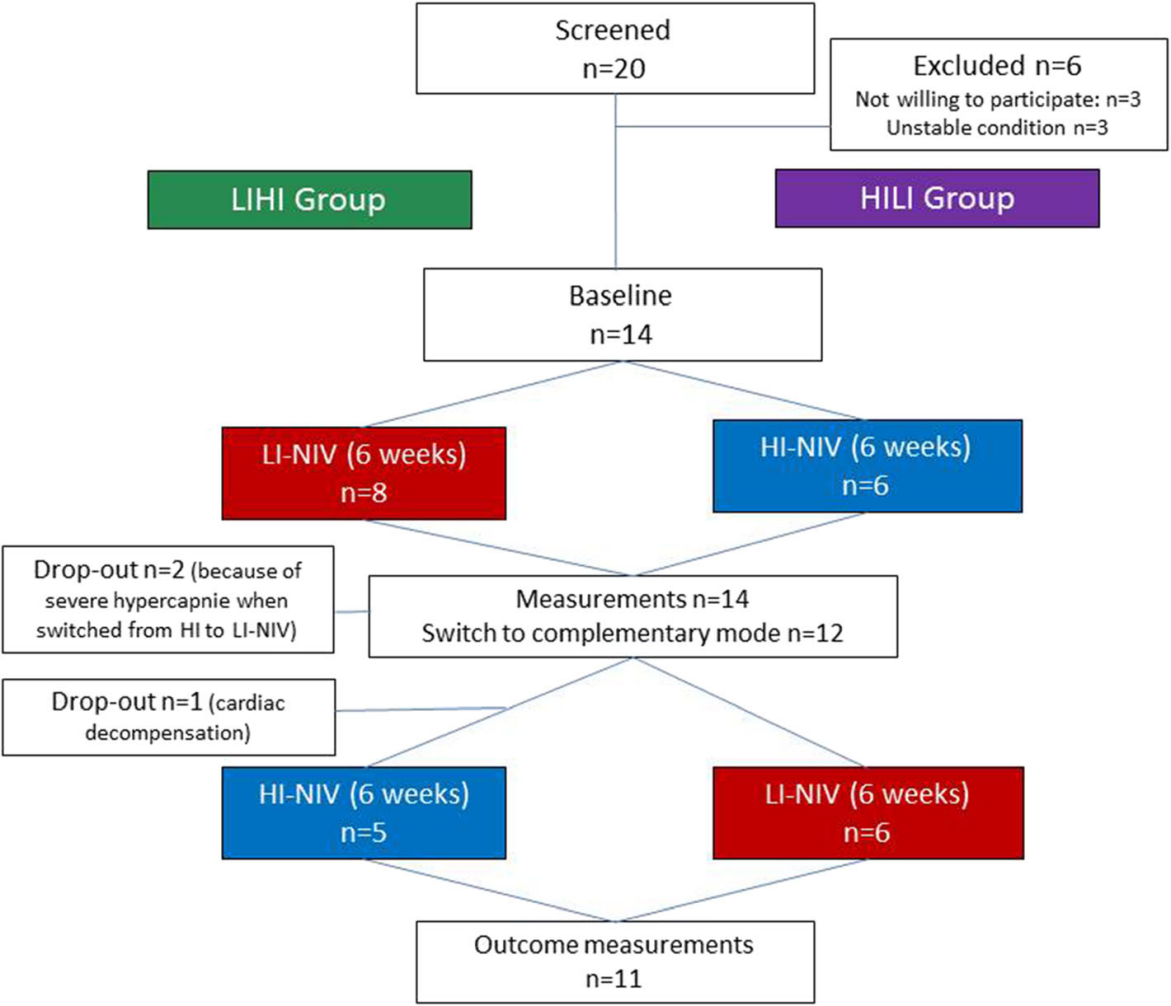
Study flow chart. As the study was a randomized cross-over trial, patients used the LI-NIV for 6 weeks followed by HI-NIV for 6 weeks (LIHI group, *green*) or HI-NIV for 6 weeks followed by LI-NIV for 6 weeks (HILI group, *purple*)

Fourteen patients were included and 11 completed the two periods (Fig. 1). Two patients (subject ID 12 and 13) did not tolerate the change from HI-NIV to LI-NIV because PaCO_2_ increased up to 10 kPa and pH decreased below 7.35. One patient with pre-existing heart failure and atrial fibrillation (LVEF at baseline: 25%) presented with decompensated heart failure after 5 weeks of HI-NIV (subject ID 7). Coronary angiography revealed a main coronary artery stenosis. After treatment, echocardiography showed no changes in right or left ventricular function, both during spontaneous breathing and under HI-NIV (Table 3). The relatively higher ventricular rate (87/min versus 68/min) during HI-NIV compared to the LI period may have provoked the cardiac decompensation.

**Table 3:** Echocardiography in the 11 patients included in the data set analysis

Patient ID	SV (ml)	CO (l/min)	CI (l/min/m^2^)	LV diastolic	sPAP (mmHg)	RVb (cm)	FAC (%)	TAPSE (mm)	S` (cm/s)
**1**									
Baseline	53	5.3	2.3	normal (E/A 0.8)	33	4.2	59	14	10
LI-NIVSB, (NIV)	63 (57)	5.1 (5.7)	2.2 (2.1)		na	4.3	55	18	10
HI-NIV SB (NIV)	87 (74)	7.0 (5.6)	3.1 (2.4)		na	4.0	47	17	15
**2**									
Baseline	56	4.6	2.7	normal (E/A 0.9)	na	3.6	44	16	11
LI-NIV SB (NIV)	72	5.5 (5.9)	3.2 (3.4)		na	3.7	50	11	12
HI-NIV SB (NIV)	65	4.8 (6.3)	2.8 (3.6)		na	4.2	45	15	17
***3***									
*Baseline*	*54*	*4.9*	*2.8*	normal (E/A 1*.*2)	*na*	*3.9*	*57*	*16*	*15*
*HI-NIV SB (NIV)*	*64 (56)*	*5.9 (6.2)*	*3.3 (3.5)*		*na*	*3.5*	*60*	*21*	*10*
*LI-NIV SB (NIV)*	*63 (72)*	*5.3 (6.3)*	*3.0 (3.6)*		*na*	*3.0*	*50*	*17*	*11*
**4**									
Baseline	72	6.9	3.8	normal (E/A 1.1)	na	2.5	70	16	17
LI-NIV SB (NIV)	66 (68)	5.8 (5.3)	3.3 (3.1)		na	3.2	66	19	10
HI-NIV SB (NIV)	73 (75)	7.5 (7.7)	4.3 (4.4)		na	na	na	19	12
**5**									
Baseline	53	4.4	1.8	restrictive,MV E Vmax 120 cm/s	na	5.4	41	20	na
HI-NIV SB (NIV)	77 (89)	4.9 (5.6)	2.0 (2.3)		29	3.7	20	15	10
LI-NIV SB (NIV)	104 (75)	7.8 (6.5)	3.2 (2.7)		18	4.2	na	18	na
**6**									
Baseline	60	5.0	2.9	normal (E/A 0.7)	43	4.2	50	20	10
HI-NIV SB (NIV)	56 (na)	4.0 (na)	2.3 (na)		23	4.2	53	23	14
LI-NIV SB (NIV)	92 (na)	6.0 (na)	3.4 (na)		44	4.1	45	22	11
***7***									
*Baseline*	*43*	*4.3*	*2.4*	MV E Vmax 80 cm/s	*46*	*4.2*		*11*	*8*
*LI-NIV SB (NIV)*	*50 (57)*	*3.4 (4.1)*	*1.9 (2.3)*		*37*	*3.8*		*11*	*na*
*HI-NIV SB (NIV)*	*45 (50)*	*3.9 (4.5)*	*2.2 (2.5)*		*31*	*4.5*		*13*	*10*
**8**									
Baseline	67	5.7	3,9	normal (E/A 0.8)	33	2.7	45	17	12
LI-NIV SB (NIV)	66 (68)	5.2 (5.2)	3.5 (3.5)		36	2.9	50	17	18
HI-NIV SB (NIV)	56 (52)	4.6 (4.2)	3.2 (2.9)		36	2.9	44	21	10
**9**									
Baseline	90	7.6	4,8	normal (E/A 0.9)	na	na	na	na	na
LI-NIV SB (NIV)	85 (74)	6.9 (5.3)	4.4 (3.4)		26	4.2	41	18	16
HI-NIV SB (NIV)	76 (67)	6.2 (5.5)	3.9 (3.5)		30	3.7	59	19	14
***10***									
*Baseline*	*79*	*6.3*	*3.8*	normal (E/A 0*.*9)	*na*	*3.4*	*70*	*15*	*16*
*HI-NIV SB (NIV)*	*84 (69)*	*4.9 (4.1)*	*2.8 (2.3)*		*na*	*3.9*	*na*	*21*	*10*
*LI-NIV SB (NIV)*	*86 (83)*	*5.4 (5.3)*	*3.1 (3.0)*		*23*	*3.6*	*48*	*16*	*10*
***11***									
*Baseline*	*78*	*4.9*	*2.9*	normal (E/A 0*.*7)	*34*	*3.2*	*60*	*15*	*15*
*HI-NIV SB (NIV)*	*52 (57)*	*3.0 (3.4)*	*1.8 (2.0)*		*na*	*3.8*	*40*	*23*	*12*
*LI-NIV SB (NIV)*	*59 (58)*	*4.8 (4.0)*	*2.9 (2.4)*		*25*	*3.7*	*65*	*25*	*12*
***12***									
*Baseline*	*86*	*6.8*	*3.8*	normal (E/A 0.9)	*24*	*4.4*		*20*	*12*
*HI-NIV SB (NIV)*	*81 (68)*	*6.9 (6.4)*	*3.8 (3.6)*		*21*	*4.1*		*25*	*18*
***13***									
*Baseline*	*56*	*4.8*	*3.3*	normal (E/A 1.4)	*39*	*2.5*		*13*	*16*
*HI-NIV SB (NIV)*	*47 (35)*	*4.1 (3.1)*	*2.9 (2.2)*		*43*	*3.1*		*21*	*11*
**14**									
Baseline	68	4.8	3.4	normal (E/A 0.7)	na	3.1	47	18	16
LI-NIV SB (NIV)	61 (60)	3.5 (3.4)	2.5 (2.4)		29	3.0	50	18	12
HI-NIV SB (NIV)	70 (71)	5.3 (4.7)	3.7 (3.3)		28	2.8	52	14	14

Individual data are shown from baseline and during spontaneous breathing (SB) after LI-NIV or HI-NIV; the values obtained during NIV are in brackets. Regular fonts are used in the order of LI-NIV followed by HI-NIV (LIHI), italic fonts in the order of HI-NIV followed by LI-NIV (HILI)

*SV* Stroke Volume (millilitres (ml)), *CO* cardiac output (litres/min (l/min), *CI* Cardiac index (l/min/m^2^ body surface area), *E/A ratio* ratio between early and late filling (E- and A-wave) of the LV: MV E Vmax: maximum velocity of the E-wave of mitral valve inflow, *sPAP* systolic pulmonary artery pressure, *RVb* basal diameter of the right ventricle, *FAC* fractional area change, *TAPSE* tricuspid annular plane systolic excursion, *S´* the pulsed Doppler peak velocity at the tricuspid annulus, *na* not applicable

Patients with ID nr. 7, 12 and 13 (italics) dropped out and were therefore not included in the data analysis

### Feasibility of cardiac output measurements in our patients

Despite limitations of echocardiography in COPD patients, the assessments of the left ventricle including the measurements of its LVOT and stroke volume to calculate CO was possible in a all patients during spontaneous breathing and in 92% of the echocardiography’s (24 out of 26 echocardiography’s) during NIV. Tachycardia due to AF occurred in one patient; moderate or severe aortic regurgitation was not present in our patients. In contrast to the left ventricle, the assessment of the right ventricle was markedly more difficult due to the poor image quality.

### Echocardiographic abnormalities at baseline

LV hypertrophy was observed in three patients. Six/13 patients, in whom the RV could be visualized, had RV enlargement (RV basal diameter >4.1 cm and or RV mid diameter > 2.7 cm), and in six patients the RV wall was thickened (>0.5 cm) [18]. All patients had normal FAC (>35%), while four patients had a mildly reduced TAPSE and/or S´. Transtricuspid flow could be detected in seven patients, while sex of them had a minimal insufficiency jet and sPAP could be measured; however, only three patients showed an abnormal value >35 mmHg (Table 3).

### Cardiac performance (completers)

**Fig. 2:**
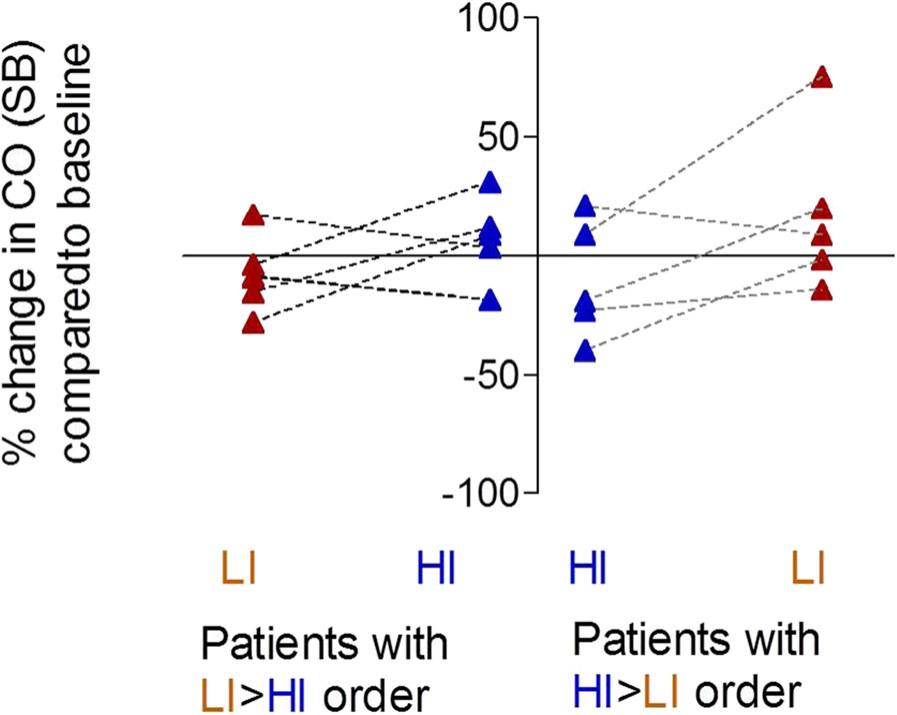
Individual changes in cardiac output (CO) during spontaneous breathing (SB) after 6 weeks NIV. Individual changes in cardiac output (CO) after 6 weeks LI-NIV are shown in *red* and 6 weeks HI-NIV are shown in *blue*. Data are shown from the 11 patients included in the data set analysis and presented in two groups: (i) Patients that started with LI-NIV followed by HI-NIV (the LIHI group, *shown left*), and (ii) patients that started with HI-NIV followed by LI-NIV (the HILI group, *shown right*)

**Fig. 3:**
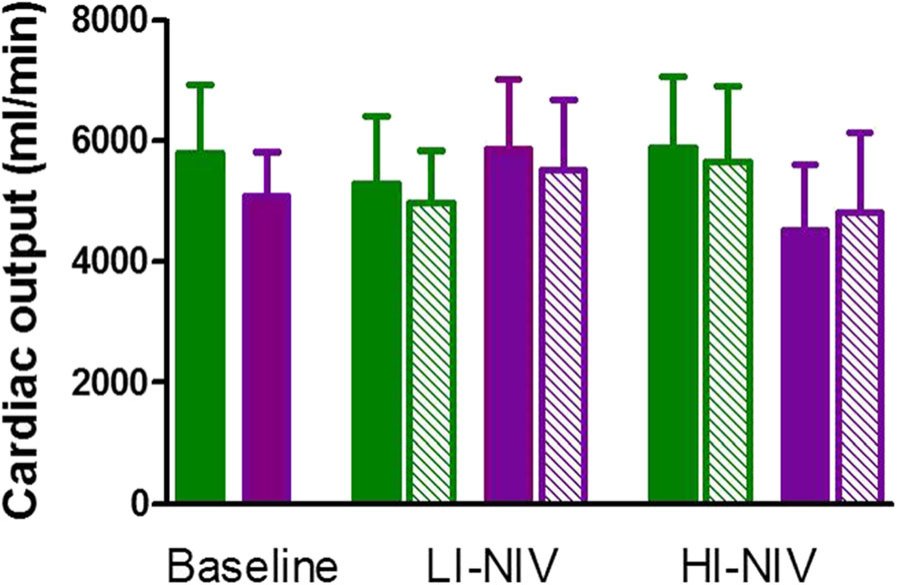
Cardiac output at baseline, after 6 weeks NIV during spontaneous breathing and under NIV. Cardiac output during spontaneous breathing is shown in the filled areas and under NIV in the dashed areas. Data are presented in two groups: (i) patients that started with LI-NIV followed by HI-NIV are shown in *green*, and (ii) patients that started with HI-NIV followed by LI-NIV are shown in *purple*

In general, 6 weeks of NIV did not affect CO during spontaneous breathing (Tables 3 and 4), although individual differences in the CO response were observed (Figs. 2 and 3 and Table 3). There was no difference in CO change between the two periods (Table 4).

**Table 4:** Outcome in the study completers after 6 weeks of NIV

Variable	Order	Baseline	LI-NIV	HI-NIV	Treatment effect HI vs. LI Mean (95% CI)	*P*
Change in CO, % of baseline	LIHI		−7.8 ± 14.9	3.2 ± 19.2	−8.5% (−27 to 10%)	0.33
	HILI		17.7 ± 34.6	−10.3 ± 24.8		
CO, L/min	LIHI	5.8 ± 1.1	5.3 ± 1.1	5.9 ± 1.2	−0.38 (−1.31 to 0.56)	0.39
	HILI	5.1 ± 0.7	5.9 ± 1.1	4.5 ± 1.1		
NTproBNP,pg/ml^##^	LIHI	177 (117–317)	113 (63–197)	158 (67–208)	37 (−26 to 101)	0.22
	HILI	215 (61–469)	199 (91–430)	158 (88–620)		
PaCO_2_, day, kPa	LIHI	6.7 ± 0.7	6.3 ± 0.7	6.1 ± 0.8*	−2.8 (−6.6 to 1.0)	0.13
	HILI	7.0 ± 1.1	6.6 ± 0.7	6.0 ± 0.7*		
FEV_1_, L	LIHI	0.72 ± 0.14	0.79 ± 0.20*	0.75 ± 0.21*	−0.00 (−0.11 to 0.10)	0.93
	HILI	0.95 ± 0.55	1.10 ± 0.63*	1.13 ± 0.63*		
SRI-SS	LIHI	43.3 ± 20.2	52.2 ± 19.7*	49.8 ± 20.8*	−2.9 (−8.0 to 2.0)	0.22
	HILI	45.2 ± 13.6	55.7 ± 8.4*	52.6 ± 9.9*		

Results are presented for the patients that followed the HILI order (HILI) and the LIHI order (LIHI) seperately. Data are presented as mean ± SD^#^, median (IQR)^##^ or mean (95% CI). * P < 0.05 compared to baseline value

*CO* cardiac output, *NT-proBNP* N-terminal brain natriuretic peptide, *PaCO*_*2*_ partial arterial carbon dioxide pressure (kilopascal (kPa)), *FEV*_*1*_ forced expiratory volume in 1 s, *SRI-SS* severe respiratory insufficiency questionnaire summary score

Compared to spontaneous breathing, CO did not acutely change with the application of NIV (Table 3, Fig. 3; P > 0.05). In one patient, estimation of CO under NIV was not possible as the heart was pushed downwards and only the subxiphoidal view was detected (see Additional file 2: Video S1 for an apical 4-chamber echocardiography view under spontaneous breathing, Additional file 3; Video S2 for a subxiphoidal echocardiography view under spontaneous breathing and Additional file 4: video S3 for a subxiphoidal echocardiography view under HI-NIV).



**Additional file 2: Video S1.** Apical 4-chamber view with very good sound conditions under spontaneous breathing. (AVI 11661 kb)




**Additional file 3: Video S2.** The subxiphoidal view shows the diaphragmatic motion under spontaneous breathing. (AVI 11130 kb)




**Additional file 4: Video S3.** Under high NIV therapy, only the subxiphoidal view is found. Have a look to the diaphragm that seems to be completely flattened. (AVI 19351 kb)


The increased sPAP in one patient could only be reduced with HI-NIV (Table 3). RV dimensions did not change consistently, but normalized in the three patients with a reduced TAPSE or S’ after 6 weeks of NIV (Table 3).

No significant changes in blood pressure or rhythm disturbances were observed. NTproBNP did not change significantly after 6 weeks of either LI-NIV or HI-NIV (Table 4).

### Pulmonary function and HRQoL

Both modes of ventilation improved FEV_1_ and HRQoL. HI-NIV tended to be more effective in improving gas exchange, although the change was not significant (Table 4).

**Fig. 4:**
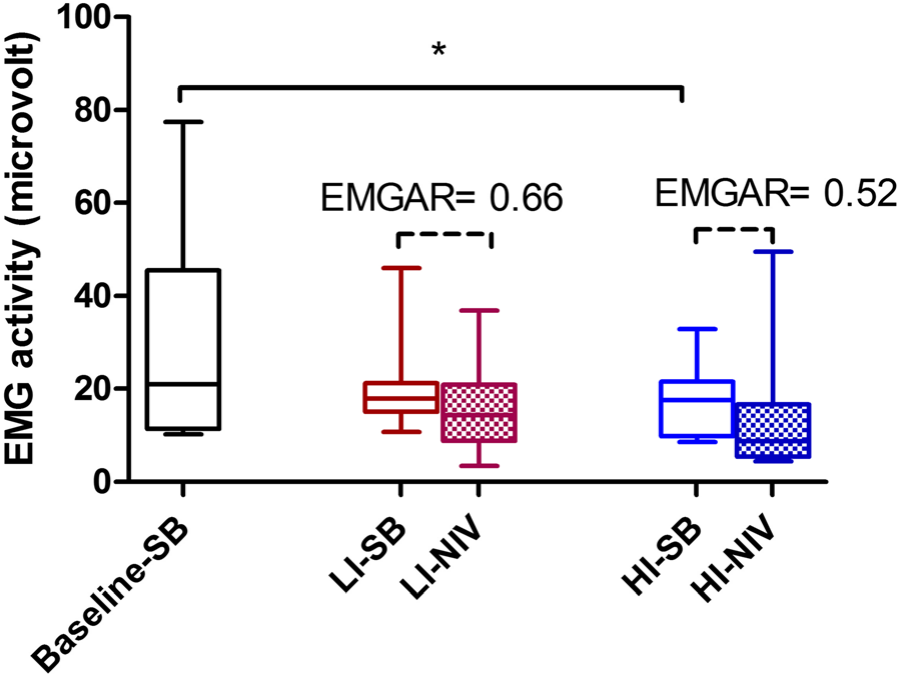
Summed EMG activity of the intercostal muscles and diaphragm during spontaneous breathing and under NIV. EMG during spontaneous breathing is shown in the filled areas and under NIV in the dashed areas. Data are presented as median (IQR). EMGAR: EMGactivity ratio: activity during NIV/activity during SB. An EMGAR of 0.66 means a 1.5 fold decrease in EMG activity under LI-NIV vs. SB, an EMGAR of 0.52 means a 1.9 fold decrease in EMG activity under HI-NIV vs. SB. The EMGARs did not differ significantly. *: P < 0.01, depicting a significant decrease in SB respiratory muscle activity after 6 W HI-NIV compared to baseline SB

Both modes tended to decrease respiratory muscle activity, and this was maintained during spontaneous breathing during the day (Fig. 4).

For other lung function parameters see Additional file 1.

## Discussion

The present pilot feasibility study shows that 6 weeks NIV in stable hypercapnic COPD has differential effects on cardiac output, depending on the patient and ventilator settings. Nevertheless, the effects of 6 weeks NIV, even HI-NIV, seem to be without clinical consequences on cardiac performance in most patients.

With echocardiography we were in most cases able to assess cardiac function quite well, although limitations were present and estimates of pulmonary hypertension were difficult to assess. Echocardiography seems to be feasible to assess cardiac function, but limitations need to be kept in mind.

Next to the cardiac outcomes, we surprisingly found that, in COPD patients with moderately-severe chronic hypercapnic respiratory failure, lower intensity NIV might be sufficient to improve HRQoL and lung function, although daytime gas exchange only improved significantly with HI-NIV.

Mechanical ventilation is known to influence cardiac performance [12–14]. While its negative impact is mainly a consequence of increased intrathoracic pressure and higher lung volumes, the effects of NIV on work of breathing, gas exchange, pulmonary vascular resistance and LV afterload might counterbalance these negative influences. Furthermore, neurohumoral mechanisms, such as the NIV-related reduction in natriuretic peptides [17] can affect body fluid balance and restore right atrium preload through an increase in mean systemic pressure. Despite our study being small and not powered on these parameters, we did find a trend towards a reduction in work of breathing and NTproBNP after 6 weeks of NIV.

We included patients known to have a heart condition. Although cardiovascular disease is a frequent comorbidity in COPD, patients with cardiac comorbidities have been purposely excluded from previous randomised controlled trials [32]. However, investigating the effect of NIV is of utmost importance especially in these patients, since the increased intrathoracic pressure induced by NIV might lead to undesirable consequences in patients with cardiac comorbidities. In our study, 3 patients with a reduced LVEF were included. Although the changes in cardiac performance amongst these patients were variable, ranging from improvement to deterioration of cardiac performance after 6 weeks NIV, these changes occurred quite constant and in the same direction with both HI-NIV and LI-NIV within each individual patient. Overall, LI-NIV seemed to be more beneficial or less harmful compared to HI-NIV in these patients.

The majority of our stable COPD patients had an enlarged RV and increased RV wall thickness. Changes in RV morphology and function in COPD are thought to occur secondarily to changes in the pulmonary vascular bed [33, 34], but might also be present long before patients meet the criteria for pulmonary hypertension. These changes are thought to be influenced not only by PaO_2,_ but also by lung hyperinflation, systemic inflammation, endothelial dysfunction and diastolic overload of the RV due to salt and water retention in chronic hypercapnic respiratory failure [35]. In our study, all patients suffered from chronic hypercapnic respiratory failure, and this might be an explanation for the prevalent changes in RV morphology.

Pulmonary hypertension was only suspected in one patient. However, echocardiography is not the gold standard for detecting PH [36]. Moreover, studies in COPD patients with right heart catheterization report huge differences in pulmonary hypertension prevalence, depending on the population, condition and treatment of the patients [37–40]. In our study, all patients had severe but stable COPD with primarily type II respiratory failure (chronic hypercapnic respiratory failure), and 11 out of 14 were normoxemic at rest while using LTOT. Both underestimation of PH because of the method used (echocardiography) and the phenotype of respiratory failure with primarily hypercapnia might eventually explain for the low incidence of pulmonary hypertension in our study. Nevertheless, changes in RV morphology, as found here in almost all patients, might preclude the development of right heart failure, which is strongly associated with worse outcomes in COPD patients.

The overall RV dimensions did not change with NIV use. Previous studies in patients with Obesity Hypoventilation Syndrome, obstructive sleep apnoea, or restrictive thoracic diseases have shown a reduction in pulmonary hypertension and RV morphology after treatment with CPAP or NIV, respectively [41–43]. However, our time frame was short and, more importantly, the degree of RV dysfunction/pulmonary hypertension was mild in our cohort.

Compared to spontaneous breathing, also during acute application of NIV, after a 6 week-period of home NIV, CO was not significantly reduced. We hypothesize that the 6-week acclimatization period allows the cardiovascular system sufficient time to adapt to the new balance. This is reassuring, as chronic NIV is supposed to have long-term benefits. However, HI-NIV treatment did reduce CO in two patients with known heart failure and in patients ventilated with very high IPAP. Furthermore, NIV changed the morphology of the thorax in one patient, although without functional consequences. These aspects thus require further investigation.

In the present study, HI-NIV was defined as the mode of ventilation necessary to achieve normocapnia; [31] mean inspiratory pressures of 23 cm H_2_O were used. This is considerably lower than the initial high-intensity trials [11], but it fits the way HI-NIV is defined as pressures needed to achieve an optimal reduction in PaCO_2_ and this is in line with the pressures used in “real life” trials [8, 9]. So, we do believe that also our results are “real-life” and generalize to the “real-life” setting. However, lower IPAP might explain the absence of (acute) hemodynamic effects of NIV in our study and it is possible that when extreme high IPAP levels are necessary to improve gas exchange, cardiac output might be depressed.

Nevertheless, even lower NIV intensities were sufficient to improve patients´ HRQoL and lung function in our patients with modest initial hypercapnia. This emphasizes the fact that beyond the use of particular IPAP, proper and individualized NIV titration should be performed to provide an optimal therapy. Clinical outcomes should guide this tailored process to each patient in the heterogeneous collective of ventilated patients.

A limitation of our study is that we included a small number of patients, of which three dropped out. As a consequence, only 11 patients were included in our full data set analysis, while our power analysis revealed that we should have included at least 12 patients in order to detect a clinically relevant change of at least 10% in CO between LI-NIV and HI-NIV compared to baseline. This might result in differences in changes between the settings not being statistically significant because we did not include enough patients. For our primary outcome (change in CO), however, results were so diverse that this is not expected to change our conclusions for now. However, a larger study should confirm the results found in our pilot. Secondly, another limitation is that we included a group of patients with different kind of cardiac comorbidities. Because sufficient knowledge in COPD patients with cardiac comorbidities is very limited, this pilot trial first intended to include patients with a broad spectrum of cardiac comorbidities, without further inclusion limits to specific comorbidities. This strategy resulted in heterogeneity of comorbidities, which makes it extremely important to judge the data not only in terms of means but also in terms of individual patient outcomes. Therefore, we provided individual patient data. Thirdly, we used echocardiography to test cardiac function. Especially in patients with COPD this method has its drawbacks and echo-windows might be insufficient to make reliable measurements. To optimize the measurements, all the echocardiography’s were performed by the same experienced cardiologist blinded to the NIV mode. With this approach, we succeeded to measure CO with Simpson’s rule in all patients. However, PH estimates were difficult and it is also known that echocardiography is not the standard method to diagnose PH. However, echocardiography’s are non-invasive and do not give discomfort to these already very disabled patients. We suggest that further studies should both include echocardiography’s and probably more invasive or uncomfortable measurements, such as cardiac MRI, to judge cardiac function under NIV. Finally, to avoid the problems associated with multiple testing with a limited amount of patients, we restricted our analyses to our main outcomes.

## Conclusions

Long-term home NIV with adequate pressure to improve gas exchange and HRQoL has differential effects on cardiac output, depending on the patient and ventilator settings. Although the treatment effects found in our pilot study should be used cautiously, in general, our preliminary data suggest that there is no reason to withhold COPD patients from HI-NIV due to concerns of adverse cardiac outcomes. Nevertheless, care should be taken in patients with co-morbid heart failure and their heart function should be checked regularly. Future trials should be performed in specific patient groups, including those with co-existing cardiac diseases.

## Additional files

Additional file 1: Supplement to Methods and Results. (DOCX 41 kb)

## Abbreviations

BMI: Body mass index
BURR: Back-up respiratory rate
CAT: COPD assessment test
CI: Cardiac index
CO: Cardiac output
COPD: Chronic obstructive pulmonary disease
EMG: Electromyography
EPAP: Expiratory positive airway pressure
FAC: Fractional area change
FEV_1_: Forced expiratory volume in 1 second
FVC: Forced vital capacity
HCO_3_^−^: Bicarbonate
HILI: HI-NIV followed by LI-NIV
HI-NIV: High-intensity noninvasive ventilation
HR: Heart rate
HRQoL: Health-related quality of life
IPAP: Inspiratory positive airway pressures
kPa: Kilopascal
LIHI: LI-NIV followed by HI-NIV
LI-NIV: Low-intensity-NIV
LV: Left ventricle
LVEF: Left ventricle ejection fraction
na: Not applicable
NIV: Non-invasive ventilation
NTproBNP: N-terminal brain natriuretic peptide
PaCO_2_: Arterial carbon dioxide
PaO_2_: Partial arterial oxygen pressure
RV: Right ventricle
RV/TLC: Residual volume as a percentage of total lung capacity.
RVb: Basal diameter of the right ventricle
S´: The pulsed Doppler peak velocity at the tricuspid annulus
sPAP: Systolic pulmonary artery pressure
SRI: Severe Respiratory Insufficiency Questionnaire
SRI-SS: Severe respiratory insufficiency questionnaire summary score.
SV: Stroke volume
TAPSE: Tricuspid annular plane systolic excursion
