# Supplementary material for: Impact of High-Intensity-NIV on the heart in stable COPD: a randomised cross-over pilot study
Source: Respir Res. 2017 May 2;18:76. doi: 10.1186/s12931-017-0542-9 (PMC5414301; doi:10.1186/s12931-017-0542-9)
Supplement: Supplementary file 4 — Supplement to Methods and Results. (DOCX 41 kb) [file 12931_2017_542_MOESM1_ESM.docx]

**Impact of High-Intensity-NIV on the heart in stable COPD: A randomized cross-over pilot study**

Running title: Cardiac impact of 6-weeks NIV in stable COPD

Marieke L. Duiverman^1,2,3*^, MD PhD; Petra Maagh^4*^, MD PhD; Friederike Sophie Magnet^3^, MD PhD; Claudia Schmoor^5^, PhD; Maria P. Arellano-Maric^3,6^, MD; Axel Meissner^4^, MD PhD; Jan Hendrik Storre^3,7^, MD PhD; Peter J. Wijkstra^1,2^, MD PhD; Wolfram Windisch^3^, MD PhD; Jens Callegari^3,^ MD PhD.

*The first and second author contributed equally.

^1^Department of Pulmonary Diseases, University Medical Center Groningen, University of Groningen, Groningen, The Netherlands.

^2^Groningen Research Institute of Asthma and COPD (GRIAC), University of Groningen, Groningen, The Netherlands.

^3^Cologne Merheim Hospital, Department of Pneumology, Kliniken der Stadt Köln gGmbH Witten/Herdecke University, Faculty of Health/School of Medicine

^4^Department of Cardiology, Kliniken der Stadt Köln gGmbH, Köln, Germany

^5^Clinical Trials Unit, Faculty of Medicine and Medical Center - University of Freiburg, Germany.

^6^Department of Pulmonary Diseases, Pontificia Universidad Católica de Chile, Santiago, Chile

^7^Department of Pneumology, University Medical Hospital, Freiburg, Germany

Additional File 1

**Abbreviations**

CO_2_ : carbon dioxide

FEV_1_: forced expiratory volume in 1 second

V_T_/t_i_ : inspiratory flow rate

t_i_: inspiratory time

P_I_max: maximal inspiratory pressure

NIV: noninvasive ventilation

P0.1: Occlusion pressure

PaCO_2_: partial arterial carbon dioxide pressure

V_T_: tidal volume

t_tot_: total duty cycle time

***Methods***

***Pulmonary outcomes***

Gas exchange was monitored by: (i) arterial blood gases taken from the arterialized earlobe (ABL 800 flex^®^ (Firma Radiometer, Dänemark) during the day without NIV, and at night during sleep (at baseline during spontaneous breathing, and after 6 weeks of NIV), and (ii) transcutaneous measurements of CO_2_ (SenTec Digital Monitoring System; SenTec AG; MPB-Software^®^: V05.03.02, SMB-Software^®^: V07.03.1; SenTec AG; Therwil, Switzerland)).^1, 2^ Blood gases were taken with the regular amount of oxygen prescribed to the patient.

Lung function (spirometry, bodyplethysmography and respiratory muscle testing) was assessed according to international guidelines.^3-5^ Daytime breathing patterns during spontaneous breathing were assessed by instructing the patients to breath quietly for at least 2 minutes through a mouthpiece connected to a pneumotachograph. Tidal volume (V_T_) was obtained by integration of the flow signal. Breathing patterns were analysed from the flow signal. Respiratory timing was analysed using the inspiratory time (t_i_)/total duty cycle time (t_tot_) ratio. The mean inspiratory flow was calculated by dividing the VT by the t_i_. Occlusion pressure (P0.1) was measured at least 6 times, and the median value of these was used for analysis. Maximal inspiratory pressure (P_I_max) was measured at least 5 times, and the highest value with a difference <10% was used for analysis. Effective inspiratory impedance was calculated by diving the P0.1 by the average inspiratory flow rate (V_T_/t_i_).

The P0.1/P_I_max ratio was used to assess respiratory drive. Furthermore, respiratory neural drive was assessed with a surface electromyography (DEMCON - Macawi Respiratory Systems BV, Enschede, the Netherlands) method, as previously described.^6^ Activity of the diaphragm and the intercostal muscles was measured during spontaneous breathing at baseline for 10 minutes of resting breathing, and this measurement was repeated after the 6-week periods for both 10 minutes of resting breathing and 10 minutes of NIV. For analysis of these signals, the EMG activity of the intercostal muscles and diaphragm were summed. EMG activity during spontaneous breathing (SB) was compared between baseline and after 6W LI- or HI-NIV. Furthermore, to quantify the reduction in respiratory muscle activity with either LI-NIV or HI-NIV, the EMG activity ratio was calculated: this is the ratio between the EMG activity during NIV compared to the EMG activity during the immediately-preceding period of resting breathing. This ratio was calculated per breath (mean activity of the respiratory muscle per breath (n=10 resting breaths)/mean of 10 breaths while on NIV) and per minute (sum of activity over 1 min resting breathing/sum of activity over 1 min NIV). In this context, the ratio depicts the increase or decrease of the respiratory neural drive with the different modes of NIV.

Exercise capacity was assessed by a standardised 6-minute walking test.^7^ Dyspnoea before and after the walking test was assessed by the Borg dyspnoea scale.

***NIV settings***

For patient tolerance, a variable inspiratory time of 0.5-1.5 seconds was allowed during both ventilation modes. The quickest rise in pressure was chosen for all patients on both settings. The inspiratory flow trigger was set to 3 l/min for both HI-NPPV and LI-NPPV, and the expiratory trigger was set to 70% of maximal inspiratory flow. The ventilators VIVO 50^®^ (Breas Medical AB, Molnlycke, Sweden) and Stellar 100^®^ (Resmed, Martinsried, Germany) were used. Commercially-available nasal masks (in 3 patients) (Air Fit Nasal^®^, Resmed, Germany) or oronasal masks (in 8 patients) (Quattro FX, Resmed, Germany; or Flexifit Fullface mask^®^, Fisher and Paykel, Germany) were used. Passive leakage circuit systems with a vented mask or an active valve system were used to prevent CO_2_ rebreathing. Oxygen was supplied in order to achieve an oxygen saturation level above 92% whilst on NIV

**Results**

In one patient, echocardiographic estimation of CO under NIV was not possible as the heart was pushed downwards towards a flattened diaphragm. Only the subxiphoidal view was detected. Video’s are shown online.

Video S1 (Additional File 2):

Apical 4-chamber view with very good sound conditions under spontaneous breathing.

Video S2 (Additional File 3):

The subxiphoidal view shows the diaphragmatic motion under spontaneous breathing.

Video S3 (Additional File 4):

Under high NIV therapy, only the subxiphoidal view is found. Have a look to the diaphragm that seems to be completely flattened.

***Gas exchange***

Both modes improved gas exchange significantly (e-Table 1), however, the difference in daytime PaCO_2_ change was not significant between the two periods (-2.8 kPa (95% CI -6.6 to 1.0); P=0.13).

**e-Table 1**: Pulmonary parameters during spontaneous breathing after 6W LI-NIV and 6W HI-NIV.

|  |  | **Baseline** | **LI-NIV** | **HI-NIV** |
| --- | --- | --- | --- | --- |
| **PaO_2_, day, kPa** | LIHI | 9.4±1.0 | 9.7±1.3 | 9.6±1.1 |
|  | HILI | 8.2±1.5 | 9.1±1.9 | 7.8±0.9 |
| **PaCO_2_, day, kPa** | LIHI | 6.7±0.7 | 6.3±0.7 | 6.1±0.8***** |
|  | HILI | 7.0±1.1 | 6.6±0.7 | 6.0±0.7***** |
| **HCO_3_^-^, day, mmol/l** | LIHI | 28.7±1.5 | 27.2±1.8 | 28.7±3.3 |
|  | HILI | 30.5±3.6 | 28.1±1.3 | 26.5±1.5 |
| **P_t_CO2, night, kPa** | LIHI | 7.1±0.9 | 6.3±0.7* | 5.0±1.1* |
|  | HILI | 8.0±1.7 | 6.2±0.6* | 6.1±0.7* |

***e-Table 1 Legend***

The two periods represent the order of the consecutive periods (LI-NIV followed by HI-NIV (LIHI) or HI-NIV followed by LI-NIV (HILI). *: P<0.05, effect of each period in comparison to the baseline value, analysed with the ANOVA model, with correction for randomised sequence.

PaO_2_: partial arterial oxygen pressure (in kilopascal (kPa) at daytime during spontaneous breathing with the regular amount of oxygen prescribed; PaCO_2_: partial arterial carbon dioxide pressure; HCO_3_^-^: bicarbonate; P_t_CO2: mean transcutaneous carbon dioxide pressure over the night, drift-corrected values were used.

To avoid the problems associated with multiple testing in this small group, the differences in secondary parameter changes between ventilation periods were not tested for statistical significance in these groups. These data should therefore be regarded as descriptive.

***Lung Function, breathing patterns and exercise tolerance***

FEV_1_ improved with both modes, without significant changes between the two periods. Lung volumes did not change. Patients increased their inspiratory time and their spontaneous breathing tidal volumes after 6W NIV. Inspiratory flow and effective inspiratory impedance did not change. Of note, the 6-minute walking distance did increase only significantly after 6 weeks LI-NIV (e-Table 2 and e-Table 3).

**e-Table 2**: Pulmonary parameters during spontaneous breathing after 6 weeks LI-NIV and 6 weeks HI-NIV.

|  |  | **Baseline** | **LI-NIV** | **HI-NIV** |
| --- | --- | --- | --- | --- |
| **FEV_1_, L** | LIHI | 0.69±0.15 | 0.79±0.20***** | 0.74±0.20***** |
|  | HILI | 0.95±0.55 | 1.10±0.63***** | 1.13±0.63***** |
| **FVC, L** | LIHI | 1.47±0.63 | 1.61±0.52 | 1.57±0.45 |
|  | HILI | 1.74±0.84 | 2.27±1.15 | 2.12±0.87 |
| **TLC,%pred** | LIHI | 102±22 | 98±20 | 103±17 |
|  | HILI | 121±33 | 119±23 | 118±23 |
| **RV, %pred** | LIHI | 171±59 | 164±54 | 179±44 |
|  | HILI | 243±87 | 214±64 | 216±64 |
| **RV%TLC** | LIHI | 67±8 | 66±5 | 70±6 |
|  | HILI | 75±14 | 68±15 | 69±13 |
| **VT, L** | LIHI | 0.58±0.12 | 0.70±0.21 | 0.60±0.13 |
|  | HILI | 0.71±0.24 | 0.87±0.34 | 0.78±0.15 |
| **BF, breaths/min** | LIHI | 22±3 | 21±5 | 22±5 |
|  | HILI | 21±4 | 17±2 | 18±2 |
| **VE, L/min** | LIHI | 12.6±1.6 | 14.1±2.1 | 12.7±3.1 |
|  | HILI | 14.8±4.2 | 14.5±6.4 | 14.5±4.0 |
| **Ti/Ttot** | LIHI | 0.37±0.06 | 0.39±0.04 | 0.41±0.04* |
|  | HILI | 0.41±0.06 | 0.40±0.06 | 0.42±0.06* |
| **VT/Ti** | LIHI | 0.60±0.10 | 0.61±0.14 | 0.57±0.13 |
|  | HILI | 0.60±0.16 | 0.61±0.21 | 0.58±0.15 |
| **6-mwd** | LIHI | 182±109 | 227±113 | 213±105 |
|  | HILI | 248±146 | 278±109 | 266±125 |

***e-Table 2 Legend***

The two periods represent the order of the consecutive periods (LI-NIV followed by HI-NIV (LIHI) or HI-NIV followed by LI-NIV (HILI). *: P<0.05, effect of each period in comparison to the baseline value, analysed with an ANOVA model, with correction for randomised sequence.

FEV_1_: forced expiratory volume in 1 second; FVC: forced vital capacity; TLC: total lung capacity, % of predicted; RV: residual volume, % of predicted; V_T_: tidal volume and BF: breathing frequency, during spontaneous breathing at rest; 6-mwd: 6-minute walking distance.

**e-Table 3**: Respiratory muscle function and neural drive

|  |  | **Baseline** | **LI-NIV** | **HI-NIV** |
| --- | --- | --- | --- | --- |
| **P_I_max** | LIHI | 3.9±1.3 | 4.6±2.8 | 5.0±2.4 |
|  | HILI | 2.3±0.9 | 4.6±2.7 | 3.9±2.1 |
| **P0.1/P_I_max** | LIHI | 10 (5-19) | 10 (3-55) | 9 (3-19) |
|  | HILI | 15 (4-24) | 6 (3-8) | 12 (5-16) |
| **P0.1/V_T_/ti** | LIHI | 0.66±0.27 | 0.75±0.43 | 0.59±0.31 |
|  | HILI | 0.54±0.33 | 0.43±0.25 | 0.71±0.36 |
| **Psniff** | LIHI | 3.0 2.1 | 2.6±1.2 | 2.7 1.9 |
|  | HILI | 1.6±1.0 | 3.4±1.5 | 2.9±1.4 |

***e-Table 3 Legend***

The two periods represent the order of the consecutive periods (LI-NIV followed by HI-NIV (LIHI) or HI-NIV followed by LI-NIV (HILI)). *: P<0.05, effect of each period in comparison to the baseline value.

P_I_max: maximal inspiratory pressure; P0.1/P_I_max: ratio between the occlusion pressure and the P_I_max; P0.1/(V_T_/ti): effective inspiratory impedance: occlusion pressure/( V_T_/inspiratory time (ti)); Psniff: sniff pressure.

To avoid the problems associated with multiple testing in this small group, the differences in secondary parameter changes between ventilation periods were not tested for statistical significance in these groups. These data should therefore be regarded as descriptive.

To avoid the problems associated with multiple testing in this small group, the differences in secondary parameter changes between ventilation periods were not tested for statistical significance in these groups. These data should therefore be regarded as descriptive.

***HRQoL***

Both modes improved the SRI scores and CAT scores, without differences between the two groups *(e-Table 4)*.

***e-Table 4: Health-related quality of life***

|  |  | **Baseline** | **LI-NIV** | **HI-NIV** |
| --- | --- | --- | --- | --- |
| **SRI-RC** | LIHI | 40.6±23.3 | 59.9±15.4 | 53.6±24.2 |
|  | HILI | 37.5±15.6 | 50.6±12.2 | 46.3±5.1 |
| **SRI-PF** | LIHI | 25.8±13.9 | 36.8±30.3 | 36.8±23.5 |
|  | HILI | 27.1±25.9 | 40.0±2.3 | 36.7±10.4 |
| **SRI-AS** | LIHI | 39.3±19.7 | 62.5±18.9 | 53.6±27.8 |
|  | HILI | 46.4±18.8 | 54.3±8.1 | 48.6±11.7 |
| **SRI-SR** | LIHI | 61.8±15.9 | 57.6±24.8 | 66.7±18.6 |
|  | HILI | 80.0±16.5 | 70.8±21.2 | 77.5±17.3 |
| **SRI-AX** | LIHI | 30.8±25.0 | 55.8±19.9 | 41.7±28.2 |
|  | HILI | 27.0±36.1 | 56.0±31.1 | 48.0±35.4 |
| **SRI-WB** | LIHI | 49.1±23.4 | 48.6±25.9 | 47.6±19.6 |
|  | HILI | 54.4±28.5 | 60.0±16.7 | 61.1±23.4 |
| **SRI-SF** | LIHI | 47.3±25.6 | 46.5±27.8 | 48.4±29.4 |
|  | HILI | 52.5±18.0 | 58.2±17.4 | 50.0±16.7 |
| **CAT** | LIHI | 25.0± 8.7 | 20.7 ±5.4 | 23.2± 4.9 |
|  | HILI | 26.4± 5.6 | 23.0 ±3.2 | 21.8± 6.3 |

**e-Table 4 Legend**

The Severe Respiratory Insufficiency questionnaire (SRI) contains 7 domains: the SRI-RC: respiratory complaints; PF: physical functioning; AS: attendant symptoms and sleep; SR: social relationships; AX: anxiety: WB: well-being; SF: social functioning domain. CAT: COPD assessment test.

To avoid the problems associated with multiple testing in this small group, the differences in secondary parameter changes between ventilation periods were not tested for statistical significance in these groups. These data should therefore be regarded as descriptive.

References

1 Magnet FS, Windisch W, Storre JH. Monitoring of pCO2 during ventilation. *Med Klin Intensivmed Notfmed*. 2016;111(3):202-207.

2 Storre JH, Steurer B, Kabitz HJ, Dreher M, Windisch W. Transcutaneous PCO2 monitoring during initiation of noninvasive ventilation. *Chest*. 2007;132(6):1810-1816.

3 Wanger J, Clausen JL, Coates A et al. Standardisation of the measurement of lung volumes. *Eur Respir J*. 2005;26(3):511-522.

4 Miller MR, Hankinson J, Brusasco V et al. Standardisation of spirometry. *Eur Respir J*. 2005;26(2):319-338.

5 American Thoracic Society/European Respiratory Society. ATS/ERS Statement on respiratory muscle testing. *Am J Respir Crit Care Med*. 2002;166(4):518-624.

6 Duiverman ML, van Eykern LA, Vennik PW, Koeter GH, Maarsingh EJ, Wijkstra PJ. Reproducibility and responsiveness of a noninvasive EMG technique of the respiratory muscles in COPD patients and in healthy subjects. *J Appl Physiol (1985)*. 2004;96(5):1723-1729.

7 Anonymous Surveillance for respiratory hazards in the occupational setting [American Thoracic Society. *Am Rev Respir Dis*. 1982;126(5):952-956.
